# HLA II class alleles in juvenile idiopathic arthritis patients with and without temporomandibular joint arthritis

**DOI:** 10.1186/s12969-016-0086-4

**Published:** 2016-04-19

**Authors:** Zane Dāvidsone, Jeļena Eglīte, Arina Lazareva, Sarmīte Dzelzīte, Ruta Šantere, Dace Bērziņa, Valda Staņēviča

**Affiliations:** Departement of Paediatrics, Riga Stradiņš University (RSU), Vienības gatve 45, LV 1004 Riga, Latvia; Riga Stradiņš University, Joint Laboratory of Clinical Immunology and Immunogenetics, Rātsupītes 5, LV-1067 Riga, Latvia; Children’s University hospital, Vienības gatve 45, LV 1004 Riga, Latvia

**Keywords:** Temporomandibular joint arthritis, Juvenile idiopathic arthritis, HLA II class alleles

## Abstract

**Background:**

Temporomandibular joint (TMJ) arthritis is seen very often (38–87 %) in children with juvenile idiopathic arthritis (JIA). With contrast enhanced magnetic resonance imaging (MRI) we can detect more cases of TMJ arthritis than ever before. Previous studies show that HLA II class alleles may have protective or risk importance in JIA subtypes. Our objective is to identify HLA II class alleles of risk and protection in JIA patients with TMJ arthritis.

**Methods:**

During the period from 2010 to 2015 MRI for TMJ was performed in 85 JIA patients who were genotyped for HLA- DRB1; DQB1 and DQA1 using RT-PCR with sequence-specific primers. As a control group, data of 100 individuals were taken from the genetic bank of RSU Joint Laboratory of Clinical Immunology and Immunogenetics. Associations of DRB1; DQB1; DQA1 alleles in patients were examined individually using the *χ*^2^ test. P-value (<0.05) and odds ratio were calculated using EPI INFO 6.0 software.

**Results:**

Out of 85 JIA patients with mean age of 13.7 ± 3.0 years (range 6.9–17.9 years), 59 (69 %) were girls and 26 (31 %) were boys. The mean duration of the disease was 3.07 ± 2.35 years (range 0.2–11.0 year). JIA subtypes were as follows: seronegative polyarthritis 51 (60 %), seropositive polyarthritis 6(7 %), oligoarthritis extended 7(8 %), oligoarthritis persistent 2 (2 %) arthritis with enthesitis 14 (17 %), undifferentiated 3 (4 %) and 2 (2 %) systemic arthritis. Two groups where separated after TMJ MRI exam: first with at least two signs of active inflammation and/or any structural damage (*n* = 62); second with no pathologic signs or with slight contrast enhancement (*n* = 23). We discovered that there are risk alleles that are found in all JIA patient’s groups (MRI positive and negative groups) versus controls such as DRB1*07:01, DQB1*03:03; DQB1*05:01. Also some protective alleles as DRB1*18:01, DQB1*06:02–8 were found in overall JIA group. Alleles DRB1*12:01, DQB1*03:01; DQA1*05:01 were found to be protective for TMJ arthrits.

**Conclusion:**

In our study there were no convincing risk alleles, but there are alleles that probably are protective for TMJ arthritis like DRB1*12:01, DQB1*03:01; DQA1*05:01.

## Background

Juvenile idiopathic arthritis (JIA) is the most common autoimmune childhood disease with a main clinical sign - chronic arthritis. Arthritis in JIA can affect any joint but the temporomandibular joint (TMJ) is particularly susceptible to damage. TMJ involvement is seen very often (38–87 %) and can lead to compromised craniomandibular function, dentofacial aestethics and morphology as micrognathia, retrognathia, pathologic occlusion and reduced mouth opening [1–4]. Contrast enhanced magnetic resonance imaging (MRI) as a golden standard for TMJ arthritis diagnostics has changed perception about the prevalence of the TMJ arthritis in JIA patients. There is data that it can be asymptomatic even in 71 % if evaluated with MRI [5, 6]; therefore it is very important to find different risk factors to identify JIA patients who need early and regular evaluation of the TMJ with MRI.

It is known that TMJ arthritis can present with such clinical symptoms as pain during the jaw movement, difficulties with chewing solid food, asymmetry with maximal mouth opening, crepitation, clicking and other; however these symptoms have high specificity but low sensitivity [1, 7]. Patients very often do not complain about TMJ problems or do not connect these complaints to JIA. Even with careful rheumatologic and orthodontic evaluation many cases of TMJ arthritis can be missed [6]. On the other hand, using only clinical symptoms it is possible to overdiagnose TMJ arthritis [8].

To find those JIA patients who need early and regular TMJ evaluation by MRI, we have to take into account not only subjective complaints and symptoms but also the type and course of the disease, it’s activity and laboratory measurements. Several risk factors for TMJ arthritis have been described such as polyarticular course of the disease, arthritis in upper extremities, younger age and higher ESR at the beginning of the disease, but HLA-B27 in previous studies was thought to be protective [1]. Other studies show that TMJ arthritis prevalence in different JIA groups is not significantly different. It is known that there are cases when TMJ arthritis can develop with no other joint involvement and no obvious laboratory or clinical risk factors can be found in these cases [3].

It is known that the course of disease, including the involvement of certain joints in case of JIA, can have genetic predisposition. HLA Class I and Class II genes are described as genetic risk factors for JIA along with PTPN22 gene and IL2RA/CD 25 gene. The combination of both general autoimmune genes and JIA-specific genes contributes to the different disease phenotypes [9]. There are reports that different HLA II class alleles are with protective or risk importance in JIA subtypes. JIA is very heterogeneous group of diseases and beside 7 subtypes of JIA, there are still significant variations, especially within the polyarticular disease. HLA typing data may provide further information for JIA subtypes and in the future it could be used as a diagnostic criterion [10]. We speculate that TMJ arthritis could be more characteristic for patients with definite HLA II class alleles.

As stated previously, it is very important to use different risk factors starting from gender, age, disease type, laboratory parameters, clinical subjective and objective signs and also genetic factors to detect JIA patients who need early and regular TMJ evaluation with MRI. It can change therapeutic tactics - local (intraarticular steroids) or systemic (addition or change of biological medication), that can prevent further damage of TMJ. The aim of our study was to identify possible HLA II class alleles of risk and protection in JIA patients with TMJ involvement*.*

## Methods

We performed the retrospective study with 85 JIA patients treated at Children’s University hospital who had a MRI exam for TMJ during the period from 2010 to 2015. All of the patients were diagnosed with different JIA types using the International League of Associations for Rheumatology (ILAR) criteria. Almost all of them had a polyarticular disease course according to the American College of Rheumatology (ACR) JIA treatment groups. Approval from Central Medical Ethics Committee of Latvia was obtained and patients’ parents and patients had signed the written consent form for participating in the study.

Most of the patients were evaluated with MRI because of subjective complains and/or objective findings from TMJ. 11 patients were without subjective complaints and/or objective signs of TMJ arthritis. TMJ MRI with contrast enhancement was done with standard T1, T2 FS (fat saturation) in coronar plane; T1, T2 sagitally; after contrast enhancement (0, 2 mmol/kg) T1 sagitally and T1 axially (8–10 min after injection).

Depending on MRI findings, patients were divided into two groups: the first group had active signs of synovitis [contrast enhancement (excluding light and symmetric contrast enhancement), effusion or pannus, bone oedema] and/or joint structure damage as flattening of the mandibular head, flattening of the fossa, osteophytes and erosions. The second group did not have any active or chronic damage findings of the TMJ arthritis or had light and symmetric contrast enhancement that can be considered as a normal finding [11]. Characteristics of these two groups where analysed using STATA program. Pearson’s correlation coefficient and Fisher’s exact test where used (*P* < 0,05). In order to obtain 80 % power of the study and to detect protective OR 0.2 with *p* < 0.05, assuming prevalence among controls 70 % and cases 30 % it was calculated that at least 36 cases and 18 controls (if Case-control ratio is 0.5) had to be included.

The immunogenetic part of the study was done at Riga Stradiņš University (RSU) Joint Laboratory of Clinical Immunology and Immunogenetics (JLCII). As a control group, samples of 100 healthy individuals from the JLCII genetic bank were utilized. The DNA was extracted from peripheral blood, by using QiagenQIAamp DNA kit reagents as per the manufacturer’s protocol [12]. The quality and quantity of DNA was checked by using *Qubit ® fluorometer* (*Invitrogen* USA). HLA typing tests and group data of healthy donors HLA genotyping was done using mutliprimer realtime polymerase chain reaction (RT-PCR) method. Patients and controls were fully genotyped for HLA- DRB1* 01:01 to 18:01 specificity, DQA1*01:01, 01:02, 01:03, 04:01, 06:01, and for DQB1*02:01-02:02, *03:01-03:05, *04:01-04:02, *05:01-05:04, and *06:01- 06:08 using Real-time PCR, qualitative analysis, melting curve analysis that is manufactured in Russia, “DNA-Technology”. Amplification was done using Real-Time PCR Thermal Cycler”DT-Lite” with four channels and 48 wells. (DNA-Technology, Russia). The reaction mixture was subjected to 35 amplification cycles, each consisting of denaturation at 94 °C (60 s), followed by one cycle, annealing at 94 °C (20 s), 67 °C (2 s) followed by seven cycles and extension at 93 °C (5 s), 65 °C (4 s), with a final extension instep with 35 cycles [13].

Associations of DRB1, DQB1 and DQA1 alleles among patient group were examined individually using the *χ*^2^ test (*P*-value <0,05). Odds ratios (OR) were calculated using EPI INFO software version 6 with 95 % confidence intervals and Fisher correction for small numbers [14].

## Results and discussion

### Results

There were 85 JIA patients with mean age of 13.7 (±3.0) years (range 6.9–17.9 years); 59 (69.4 %) girls and 26 (30.6 %) boys. The median duration of the disease before the MRI exam was 3.1(IQR 0.2–11) years. JIA subtypes were as follows: seronegative polyarthritis 51 (60 %), seropositive polyarthritis 6 (7 %), oligoarthritis extended 7 (8 %), oligoarthritis persistent 2 (2 %) arthritis with enthesitis 14 (17 %), undifferentiated 3 (4 %) and 2 (2 %) systemic arthritis patients.

After the MRI exam, we divided JIA patients into two groups – one group with TMJ arthritis findings (MRI positive group) and the other without (MRI negative group). The demographics of these two groups can be seen in the Table 1. There were statistically significant more girls in the TMJ arthritis group (*p* = 0.04). Also these patients were older - 14.2(±2.6) years (*p* = 0.01). There were no statistically significant differences between two groups in such parameters as disease duration, years from the diagnosis, active joint count and also laboratory parameters as ESR, ANA, RF and HLA-B27 antigen. While it was not statistically significant, all seropositive patients were in the TMJ arthritis positive group. Differences in CRP was statistically significant - higher CRP was in the TMJ arthritis group (*p* = 0.03).

**Table 1:** Characteristics of JIA patients with and without TMJ arthritis confirmed by MRI

Characteristics	With TMJ arthritis (*n* = 62)	Without TMJ arthritis (*n* = 23)	Total JIA (*n* = 85)
Demographics			
Gender:			
Female n (%)	47(75.8)	12(52.2)	59(69.4)
Male n (%)	15(24.2)	11(47.8)	26(30.6)
*P* value	0.04		
Mean age at the time of MRI Years (± SD)	14.2(±2.6)	12.3 (±3.6)	13.7 (±3.0)
*P* value	0.01		
Disease characteristics			
Disease duration in years	2.6(0,2–11)	3(0.6–6)	3.1(0.2–11)
Median (IQR)			
*P* value	0.45		
Years from the diagnosis	1.8(0–10)	2.2(0–7)	1.9(0–10)
Median (IQR)			
*P* value	0.07		
Active joint count^a^ Median (IQR)	7.8(0–22)	7.7(0–20)	7.8(0–22)
*P* value	0.81		
Laboratory characteristics			
ESR	12.8(2120)	6(2–20)	10.9(2–120)
Median (IQR)			
*P* value	0.07		
CRO	7.6(0180)	0.4(0–1.83)	5.6(0–180)
Median (IQR)			
*P* value	0.03		
ANA positive n (%)	8(38.1)	14(23.3)	22(27.2)
*P* value	0.19		
RF positive n (%)	6(9.8)	0(0)	6(7.1)
*P* value	0.12		
HLA B27 positive	15(24.2)	3(13.0)	18(21.2)
n (%)			
*P* value	0.25		

^a^Active joint count - joints with non-bony swelling or limitation of motion with either pain on motion or tenderness to palpation and also those where we have detected signs of arthritis using ultrasound or MRI

Our results revealed that there are general JIA risk alleles in JIA patients’ groups versus controls such as DRB1*07:01 that was found in the total JIA group and MRI positive group. Risk alleles for JIA as DQB1*03:03; DQB1*05:01 were detected in total JIA group and MRI negative group. Also some protective alleles as DRB1*18:01(in total JIA group and MRI positive group), DQB1*06:02-8 (in total JIA group and MRI negative group) were found.

Regarding TMJ arthritis alleles, DRB1*12:01, DQB1*03:01 and DQA1*05:01 appeared to be protective when comparing MRI positive versus MRI negative groups. We did not find any convincing risk alleles for TMJ arthritis (See Table 2).

**Table 2:** Comparison of HLA alleles between TMJ arthritis positive and TMJ negative patient groups and control groups

	Alleles of risk	OR (p)	Protective alleles	OR (p)
DRB1				
Total JIA group (*n* = 85) versus control (*n* = 100)	DRB1*07:01	7.28(0.000)	DRB*18:01	0.10(0.000)
TMJ arthritis positive group (*n* = 62) versus control (*n* = 100)	DRB1*13:01	1.88(0.031)	DRB1*18:01	0.13(0.001)
	DRB1*07:01	8.87(0.000)		
TMJ arthritis negative group (*n* = 23) versus control (*n* = 100)	DRB1*11:01	2.97(0.002)	-	-
TMJ positive (*n* = 62) versus TMJ negative (*n* = 23) group	-	-	DRB*12:01	0.35(0.005)
DQB1				
Total JIA group (*n* = 85) versus control (*n* = 100)	DQB1*03:03	2.03(0.052)	DQB1*06:01	0.20(0.024)
	DQB1*05:01	2.10(0.011)	DQB1*0602–8	0.56(0.028)
TMJ arthritis positive group (*n* = 62) versus control (*n* = 100)	-	-	-	-
TMJ arthritis negative group (*n* = 23) versus control (*n* = 100)	DQB1*0301	2.42(0.011)	DQB1*0602–8	0.293(0.018)
	DQB1*03:03	2.582(0.051)		
	DQB1*05:01	3.187(0.002)		
TMJ positive (*n* = 62) versus TMJ negative (*n* = 23) group	-	-	DQB1*03:01	0.41(0.017)
DQA1				
Total JIA group (*n* = 85) versus control (*n* = 100)	-	-	-	-
TMJ arthritis positive group (*n* = 62) versus control (*n* = 100)	-	-	-	-
TMJ arthritis negative group (*n* = 23) versus control (*n* = 100)	-	-	DQA1*02:01	0.163(0.046)
TMJ positive (*n* = 62) versus TMJ negative (*n* = 23) group	-	-	DQA1*05:01	0.33(0.009)

*OR* odds ratio, *p* < 0,05

### Discussion

In the study of United Kingdom children [15], DRB1*07:01 was associated with a decreased risk for persistent oligoarthritis, RF positive and negative polyarthritis and enthesitis related arthritis. Similar findings are described in Mexicans [16]. Surprisingly, our results show DRB1*07:01 as a risk allele in the total JIA patient group compared to control group and also in those with TMJ arthritis (patients with polyarticular disease course and different JIA subtypes) (OR = 7,28. *p* < 0,000). In the study of Hollenbach et al. [10], they have analysed 802 JIA patients with two most common JIA subtypes - oligoarthritis (both persistent and extended) and RF negative polyarthritis. They found several risk haplotypes regardless of clinical subtype and age of onset, including DRB1-DQA1-DQB1 haplotypes: DRB1*08:01-DQA1*04:01-DQB1*04:02 and DRB1*11:03/4-DQA1*05:01-DQB1*03:01. They also determined that protective haplotypes DRB1*15:01-DQA1*01:02-DQB1*06:02 were protective. In our study we also found DQB1*06:02-8 as a protective allele regardless of TMJ involvement. Similar results are shown in a study of JIA patients in Colombian Mestizos, where allele DQB1*06:02 was found as protective [17]. In our study allele DQB1*03:01 appears to have protective role for TMJ involvement, but in Hollenbach’s study it was protective for early onset oligoarticular-persistent subtype. There are data in Hollenbach’s study suggesting that the presence of two predisposing DRB1 alleles is associated with a significantly greater predisposition to disease than a single one. We are also planning to analyse similar data in our study. As in our results, DRB1*03 was found less frequent in JIA patients than in control group in Hungarian patients [18].

A protective role of the alleles DRB1*12:01, DQB1*03:01; DQA1*05:01 in the development of TMJ arthritis may be associated with a less aggressive disease course (e.g.,these patients had a lower CRP) and consequently less radiological damage. This trend has to be analysed in the future by evaluating patients who have these alleles more carefully and over long-term course regardless of TMJ involvement.

When analysing HLA II class alleles, we must take into account the differences of nationality and different genetic background since most of our patients are of Latvian or Russian descent. Also there should be more evaluation and discussion of the associations between ILAR groups and TMJ involvement; in this study we divided the groups into MRI-confirmed and MRI-non confirmed TMJ arthritis and could not take into account, how disease could change over time with TMJ developing in the future. Most of our patients were already adolescents with mean disease duration of 3 years and it might be that the disease course was already established.

We suspect that TMJ arthritis can be used as a prognostic feature for disease course. Identifying risk and protective HLA II class haplotypes could help to predict TMJ arthritis development in the future that in turn could help to treat and prevent TMJ joint damage in high risk patients. We will evaluate further other characteristics of the patients with TMJ involvement that could help to detect JIA patients who need early and regular evaluation with MRI. It would be important to analyse HLA II class alleles in more homogeneous groups of JIA to see if the same results about protective alleles are found.

## Conclusions

In our study there were no convincing risk alleles, but there are alleles with probable protective role for TMJ arthritis such as DRB1*12:01, DQB1*03:01; DQA1*05:01. Further analysis of different haplotypes may help us detect HLA II class alleles that can be used in the early detection of high risk for TMJ arthritis.

## Abbreviations

ACR: American College of Rheumatology
ANA: antinuclear antibodies
CRP: C-reactive protein
ESR: erythrocyte sedimentation rate
HLA: human leukocyte antigens
ILAR: International League of Associations for Rheumatology
IQR: interquartile range
JIA: juvenile idiopathic arthritis
MRI: magnetic resonance imaging
PTPN22: protein tyrosine phosphatase, non-receptor type 2
RF: rheumatoid factor
RSU: Riga Stradiņš University
RT-PCR: real time polimerase chain reaction
SD: standard deviation
TMJ: temporomandibular joint
